# An Overview of Antimicrobial Activity of Lysozyme and Its Functionality in Cheese

**DOI:** 10.3389/fnut.2022.833618

**Published:** 2022-03-09

**Authors:** Nasim Khorshidian, Elham Khanniri, Mohammad Reza Koushki, Sara Sohrabvandi, Mojtaba Yousefi

**Affiliations:** ^1^Department of Food Technology Research, National Nutrition and Food Technology Research Institute, Shahid Beheshti University of Medical Sciences, Tehran, Iran; ^2^Food Safety Research Center (Salt), Semnan University of Medical Sciences, Semnan, Iran

**Keywords:** antimicrobial enzyme, lysozyme, peptidoglycan, dairy, cheese

## Abstract

Due to the concern of consumers about the presence of synthetic preservatives, researchers and food manufacturers have recently conducted extensive research on the limited use of these preservatives and the introduction and use of natural preservatives, such as herbal extracts and essential oils, bacteriocins, and antimicrobial enzymes. Lysozyme is a natural enzyme with antimicrobial activity that has attracted considerable attention to be potentially utilized in various industries. Since lysozyme is an intrinsic component of the human immune system and has low toxicity; it could be considered as a natural antimicrobial agent for use in food and pharmaceutical industries. Lysozyme exerts antimicrobial activity against microorganisms, especially Gram-positive bacteria, by hydrolyzing 1,4-beta-linkages between N-acetylmuramic acid and N-acetylglucosamine in the cell wall. In addition, increased antimicrobial activity of lysozyme against Gram-negative bacteria could be achieved by the modification of lysozyme through physical or chemical interactions. Lysozyme is presented as a natural preservative in mammalian milk and can be utilized as a bio-preservative in dairy products, such as cheese. Both bacteria and fungi can contaminate and spoil the cheese; especially the one that is made traditionally by raw milk. Furthermore, uncontrolled and improper processes and post-pasteurization contamination can participate in the cheese contamination. Therefore, besides common preservative strategies applied in cheese production, lysozyme could be utilized alone or in combination with other preservative strategies to improve the safety of cheese. Hence, this study aimed to review the antimicrobial properties of lysozyme as natural antimicrobial enzyme and its functionality in cheese.

## Introduction

Food safety is considered as one of the most important challenges in the food industry. Microbial contamination and the presence of spoilage microorganism and especially the pathogens is known as one of the major concerns in food safety. Therefore, foodstuff production free from any microbial contamination is one of the priorities of food manufacturers and regulatory organizations (1, 2). In this regard, various thermal and non-thermal preservation strategies have been utilized to control or destroy microorganisms to ensure the safety of raw or processed food (3, 4). Furthermore, various preservatives, such as synthetic antimicrobial agents as supplementary approaches have been applied to hinder the microbial contamination of food products during processing, distribution, and storage. Although these synthetic antimicrobial agents are considered generally recognized as safe (GRAS), there has been a growing concern about using synthetic additives by consumers (5, 6). Therefore, attention has been increased to use various natural additives, such as sweeteners, coloring, antioxidants and antimicrobials agents obtained from microorganisms, plants, and animal (7, 8). Various natural antimicrobial agents, such as essential oils and herbal extract, bacteriocin, and antimicrobial enzymes have been identified (9–11).

**FIGURE 1:**
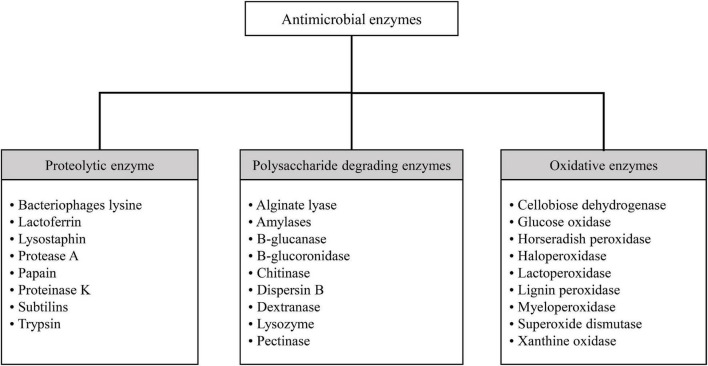
Various categories of antimicrobial enzymes.

Antimicrobial enzymes are widespread and have a key role in defending living organisms against bacterial attacks. These enzymes are now growingly used against microbial systems. As a group, they exert antimicrobial activity by directly attacking microorganisms, interfering with biofilm formation, biofilm destruction, and/or catalyzing reactions that resulted in the production of antimicrobial compounds. Different proteolytic, oxidative, and polysaccharide degrading enzymes are utilized against antimicrobial and antibiofilm systems (12). Various categories of antimicrobial enzymes are shown in Figure 1. One of these categories, as mentioned, is polysaccharide degrading enzymes, among which alginate lysase, amylase dispersin B, and lysozyme, are the most widely used.

Lysozyme, also called muramidase or N-acetylmuramic hydrolase, is a small, monomeric enzyme that hydrolyzes the β-1,4-linkages between N-acetyl-d-glucosamine and N-acetylmuramic acid residues in the peptidoglycan of bacterial cell walls (13). Lysozyme was discovered by Alexander Fleming, who accidentally recognized that a drop of his nasal mucosa caused the lysis of bacteria leading to discover a significant bacteriolytic element called lysozyme (14). Further investigations confirmed this finding, and at present, it is known that lysozyme exists in large quantities in tears, saliva, blood serum, human and cow milk, egg white of birds, and approximately in certain bacteria and bacteriophages (15–17). Lysozyme was the first enzyme that its primary amino acid sequence was characterized. Additionally, it was the first enzyme that its structure was defined by X-ray crystallography (18, 19). Lysozyme exerts antimicrobial activity against microorganisms, especially Gram-positive bacteria by hydrolyzing 1,4-beta-linkages in the bacterial cell walls. Hence this enzyme could be utilized in medical, pharmaceutical, and especially in the food industry as a natural food additives to control the bacterial growth in food products (20, 21). In the terms of availability, the chicken egg white is the most acceptable source of lysozyme. It has been widely reported that hen egg white lysozyme is generally utilized as a preservative in various products, such as fruits and vegetables, meat, milk, and dairy products (17, 22).

Since lysozyme is an intrinsic component of the human immune system, it seems that it has low toxicity in humans. It is specific for bacterial peptidoglycans, and therefore, no reaction between lysozyme and human tissue occurs (23). Since lysozyme is presented as a natural preservative in mammalian milk and due to the absorption of lysozyme by casein micelles and maintaining the activity of enzyme after absorption, this enzyme is likely to be appropriate bio-preservative in dairy products (24, 25). Among these, cheese, as one of the most popular dairy products, could be contaminated by various spoilage and pathogen microorganisms. Both bacteria and fungi can contaminate and spoil the cheese; especially the one that is made traditionally by raw milk (8, 26). Additionally, cheese could be contaminated by various microorganisms by post-pasteurization contaminants when the process is not properly controlled. *Listeria monocytogenes*, *Salmonella*, *Escherichia coli*, *Staphylococcus aureus*, and *Bacillus cereus* can be named as the most important food pathogen found in contaminated cheese (8, 27). Besides the concern about the presence of food pathogens, the spoilage of cheese may also occur, especially during ripening, such as late blowing by *Clostridium tyrobutyricum* (28). Therefore, it is required to prevent or control the microbial contamination of cheese during production, ripening, and storage. Apart from common preservative strategies applied in cheese production, there has been a growing interest in the application of natural antimicrobial agents, such as lysozyme alone or in combination with other preservative strategies (27, 29, 30). It has been stated that lysozyme could be applied to protect cheese against the development of off-flavors and curdling created by *B. cereus* and late blowing produced by *C. tyrobutyricum* (30–32). In this regard, this study aimed to review the antimicrobial activity of lysozyme as natural antimicrobial enzyme and its functionality in cheese.

## Structure, Type and Properties of Lysozyme

One of the polysaccharide-degrading enzymes is lysozyme (E.C 3.2.1.17), and its structure was first illuminated in the 1960s by X-ray crystallography (12). Lysozyme is a glycosidase (a type of murein hydrolases) with a molecular weight of almost 11–22 kDa and the pH of isoelectric point of approximately 9.5–11 (15). This enzymatic protein is known as muramidase or N-acetyl muramide glycanohydrolases. Lysozyme is found in various sources, such as all living organisms, but avian egg whites, human and cow milk, mucus, tears, and saliva are the main sources (33). Lysozyme from hen egg white (HEWL) is considered as a major industrial source for practical uses because of its availability and cost-effectiveness. There are 0.3–0.4 g of lysozyme in each chicken egg (3.5% of total egg white proteins) with maximum enzymatic activity at pH of around 5 (21, 34).

Lysozyme can split the bond between N-acetyl-D-glucosamine and N-acetyl-muramic acid residues of the peptidoglycan chains and thus act as an antimicrobial agent, especially against Gram-positive bacteria (21). In terms of its structure, lysozyme has 129 amino acids cross-linked by disulfide bridges in four different places of a single polypeptide chain. N-terminal and C-terminal amino acids in lysozyme structure are lysine and leucine, respectively (15, 35). The high thermal stability of lysozyme is due to the presence of the six helix regions and disulfide bridges in its structure. The α- and β-domain are two main domains in the polypeptide chain of lysozyme and the binding site is located between them in a large crevice. The active site consists of the amino acids of glutamic acid 35 (Glu35) and aspartate 52 (Asp52) that have important roles in lysozyme’s catalytic activities. Glu-35 gives a proton to the glycosidic oxygen, thus creating oxonium and carbenium ions and cleaving the substrate’s C–O bond. The carbenium ion interacts with the negatively charged Asp52 residue and forms the glycosyl intermediate. Then, the surrounding water provides a hydroxyl ion to combine with the carbenium ion and the lysozyme is separated from the glycosyl–enzyme intermediate. Thereby, the lysozyme molecule remains unchanged and the substrate is hydrolyzed (20).

Based on the differences in amino acid sequences and structural properties (tertiary structures), lysozymes are divided into three main types: c-type, g-type, and i-type (36). The properties of each type of lysozyme are presented in Table 1. The first type of lysozyme has been categorized as the chicken or conventional type (c-type), which human lysozyme and chicken egg-white lysozyme are related to this category. Although human lysozyme has shown higher antibacterial activity and thermal stability than lysozyme isolated from hen egg-white, its utilization has been limited due to its restricted available resources (33). The g-type lysozyme is a characteristic of goose egg-whites that its molecular weight is 21 kDa. Lysozymes of g-type were also found in rhea, cassowary, ostrich, black swan, and some fish species (37). The amount of cysteine and tryptophan is low in typical g-type lysozymes, which causes their thermal instability (38). Moreover, i-type lysozymes are the lysozymes isolated from invertebrates that are smaller than the lysozymes of g-type. The lysozymes of i-type have been identified in *Nematoda*, *Mollusca*, insects, *Crustacea*, *Echinodermata*, and *Annelida* (37). Although different types of lysozymes may be present in the same species simultaneously, they have different enzymatic and biochemical characteristics and can have different or complementary functions.

**TABLE 1:** Characteristic of the three types of lysozymes in the animal kingdom.

Lysozyme	Main sources	Amino acids in representatives lysozymes	Molecular weight (kDa)	Anti-Gram negative activity	References
c-type	Most vertebrates including mammals representative lysozyme: hen egg white (HEWL)	129	∼11–15	Lysozymes isolated from the ayu fish, coho salmon eggs, Japanese flounder and the kidney of the rainbow trout.	(20, 41)
g-type	Avian species (chicken, black swan, ostrich cassowary and rhea) representative lysozyme: goose egg white (GEWL)	185	∼20–22	Lysozymes from yellow croaker, orange-spotted grouper and Japanese flounder	(20, 75)
i-type	Invertebrates representative lysozyme: the marine bivalve *Tapes japonica* (TJL)	123	∼11–15	Lysozymes in the eastern oyster (*Crassostrea virginica*) and sea cucumber (*Stichopus japonicus*)	(76, 77)

## Antimicrobial Activity of Lysozyme

**FIGURE 2:**
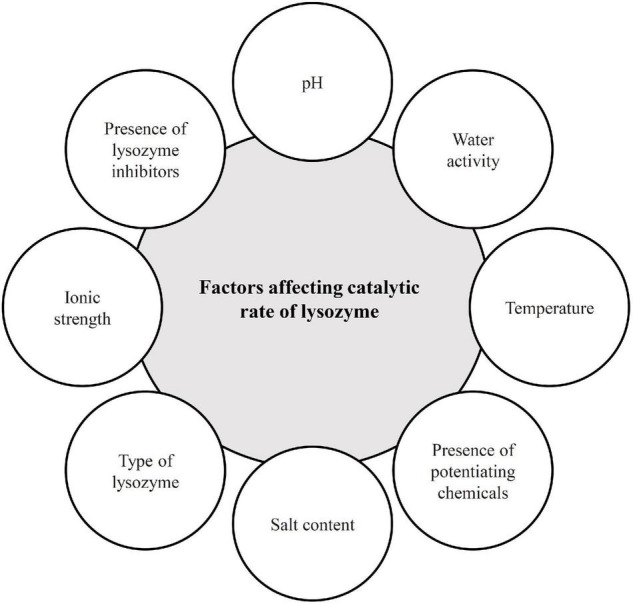
Various effective factors on the catalytic rate of lysozyme.

Lysozyme as an enzymatic globular protein has gained great attention in the pharmaceutical and food industries owing to its antibacterial function. As reported, all lysozymes can hydrolyze the β-(1,4)- glycosidic linkages between N-acetylmuramic acid (NAM) and N-acetylglucosamine (NAG) of peptidoglycan chains. Peptidoglycan specifies cellular shape and creates a good protection versus cellular turgor pressure, whereas lysozyme activity causes the loss of its integrity, bacterial cell lysis, and eventual cell death (39). The cell wall of Gram-positive bacteria consists of abandon peptidoglycan layers (about 40 layers) without any outer membrane to which polysaccharides, lipoteichoic acids, and teichoic acids are connected to peptidoglycan (40). Therefore, they are susceptible to being attacked by lysozyme. The level of teichoic acids in the cell wall of Gram-positive bacteria can affect the degree of their sensitivity to lysozymes because the negatively charged teichoic acids bind the lysozymes and reduce the enzymatic activity of lysozymes (36). Various technological and environmental factors, such as pH, temperature, salt concentration, and osmotic strength can affect the stability and antimicrobial activity of lysozyme in food and this varies based on the lysozyme types (32, 41). These effective factors on lysozyme activity have been critically reviewed by Proctor and Cunningham and Taylor et al. (23, 42). The maximum enzymatic activity of HEWL is observed at approximately pH 5 and lower stability and activity has been obtained in highly acidic (pH < 3.8) and alkaline conditions (43). The pH range of lysozyme activity is highly dependent on salt concentration. In a survey conducted on human lysozyme, the results showed that in the sodium chloride concentration of 0.05 M, lysozyme activity was greater at a wide range of pH (5–9) compared with the salt level of 0.1 M (44). Lysozyme non-specific activity can take place in the low level of salt, although high-ionic strength (0.2 M salt) inhibits its activity (41). The maximum enzymatic activity was obtained at an ionic strength of 0.1 in the presence of potassium salts (43). Garajov et al. reported that the specific activity of lysozyme increased with the decreasing concentration of sodium salts of acetate, chloride, bromide, thiocyanate, and perchlorate from 0.5 to 0.1 M (45). Due to the presence of four disulfide bonds in lysozyme structure, it was active at 100°C within 2 min at pH 4–5, but at pH 9, activity loss happened with heating for 5 min at boiling temperature (46). The temperature between 25 and 60°C had no effect on human lysozyme activity (44). However, at pH 6.2, 25% loss of the lytic activity of egg-white lysozyme was reported at 100°C within 20 min and 5% of activity was lost following treatments at 80°C for 30 min (47). Moreover, there is an evidence that lysozyme can be stored at 5°C for many years or at 30°C for several months (>6 months) without losing lytic activity in dry state. Lysozyme can be protected by polyols and sugars versus heat, because they affected hydrophobic interactions (44). Xing and Yaylayan stated that the presence of sugars could even maintain the enzymatic activity of lysozyme after milling (48). The most important factors that affect the catalytic rate of lysozyme are shown in Figure 2.

**FIGURE 3:**
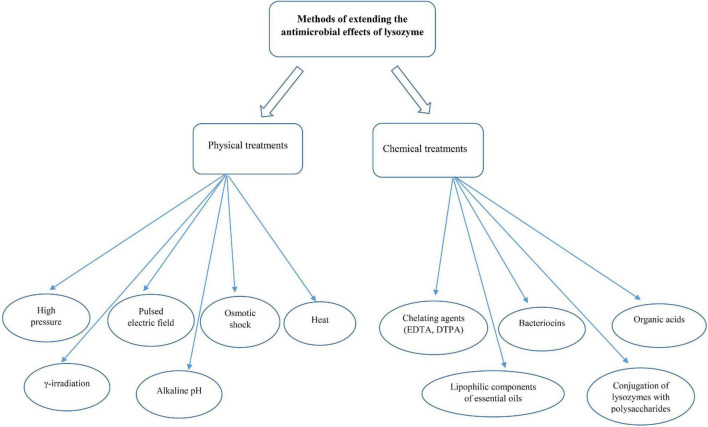
Common methods to increase the lysozyme activity against the growth of Gram-negative bacteria.

Based on the information obtained about the sensitivity of food-related bacteria to lysozyme, some Gram-positive bacteria, such as *Micrococcus* spp., *Sarcina* spp., *C. tyrobutyricum*, *Clostridium thermosaccharolyticum*, *Bacillus stearothermophilus*, and *Bacillus coagulans* were strongly lysed by egg-white lysozyme (46). On the other hand, lysozymes are ineffective against Gram-negative bacteria because they have a thin layer of peptidoglycan without teichoic acids covered by an outer membrane. The outer membrane contains phospholipids, lipopolysaccharides, and lipoproteins that act as a barrier and limit the access of lysozymes to peptidoglycan chains (36, 49, 50). Nonetheless, this barrier can be disrupted by membrane-permeabilizing agents, such as carvacrol, lactoferrin, organic acids, and ethylenediaminetetraacetic acid (EDTA) or conjugation of lysozyme to carbohydrates, denaturation of lysozyme, and the modification of lysozyme by linking with other compounds (polysaccharides, hydrophobic peptides, and fatty acids) (34). Several studies have reported that treatments, such as high hydrostatic pressure and pulsed electric field can increase the susceptibility of Gram-negative bacteria, such as *E. coli* to lysozyme (51, 52). A summary of used methods to improve the function of lysozymes against Gram-negative bacteria has been provided in Figure 3.

In addition to the enzymatic activity of lysozymes as a bactericidal action, they have non-lytic activity to kill bacteria related to their hydrophobic, structural, and cationic features. Lysozyme as a cationic protein can form pores in bacterial membranes through the electrostatic interactions between protein and components of the bacterial membrane (phospholipids) and induce cell lysis as a result of membrane leakage without peptidoglycan hydrolysis. Evidence has shown that the complete or partial denaturation of lysozyme using heat, dithiothreitol (DTT), or site-directed mutagenesis does not reduce its bactericidal effect against Gram-negative and Gram-positive bacteria due to its non-enzymatic antimicrobial properties. The bactericidal activity of denatured lysozyme is attributed to the cationic properties and the increase of hydrophobic amino acid residues on its surface owing to conformational changes (46).

Apart from the antibacterial action, lysozyme has a weak activity against yeast, because mannoprotein and fibrous β-(1,3) glucans are the main compounds of yeast cell wall and the chitinase activity of lysozyme is not strong (53, 54). Additionally, lysozyme can inhibit the growth of viruses and fungi, such as *Aspergillus niger* and *Penicillium* that is associated with its charge, rather than its enzymatic action (49). In addition, Lysozyme exhibits antiviral activity by forming complexes with the DNA of viruses (15, 37).

## Application of Lysozyme in Cheese

The high demand for natural food preservatives has made lysozyme an increasingly important natural antimicrobial enzyme in food processing and preservation. Due to the high potential of lysozyme, it could be utilized in various practical applications, such as food, pharmaceutical, and chemical industries (55, 56). As mentioned before, lysozyme as a natural antimicrobial compound can hydrolyze β-(1,4) glycosidic linkage between N-acetylmuramic acid and N acetylglucosamine found in the peptidoglycan, the main component of the cell wall of both Gram-positive and Gram-negative bacteria (57). Therefore, it can be used to inhibit bacterial growth, especially Gram-positive spoilage bacteria, in various food products, such as wine and unpasteurized beer and meat and dairy products (41, 51, 58). In the dairy industry, lysozyme could be applied in cheese making to inhibit the growth of spoilage bacteria and prevent the texture deterioration, blowing, and unpleasant taste of various cheeses (59, 60). The antimicrobial activity of lysozyme and its functionality in cheese is discussed in this part.

Bester et al. investigated the effect of various lysozyme levels on the growth of vegetative cells of *C. tyrobutyricum* and germination of its spore forms, the growth of cheese starter culture and coliform bacteria in culture media. They found that the vegetative growth of *C. tyrobutyricum* BZ15 and C611 was prevented by lysozyme at the levels equal to and greater than 250 units/ml. Furthermore, the stimulation of spores carried out in the presence of lysozyme and the maximum stimulation was observed at 250 units/ml and the spores of strain C611 exhibited faster germination in the presence of lysozyme. Three isolates of coliforms were inhibited by lysozyme at 500 and 1,000 U/ml, and only one isolated was stimulated by lysozyme. In addition, they indicated that the activity of starter cultures was not affected by various concentrations of lysozyme (up to 2,500 U/ml). They stated that the growth of four lactobacilli and the quality of the cheese obtained by these bacteria were not affected by lysozyme at levels of 250 U/ml (61). On the other hand, López-Pedemonte et al. studied the effect of the addition of nisin or lysozyme in the sensitivity of *B. cereus* ATCC 9139 to high hydrostatic pressure (HHP) in cheese and found that the sensitivity of spores to HHP was not affected by the addition of lysozyme (22.4 mg/ml), while the presence of nisin (1.56 mg/ml) significantly increased the sensitivity of the spores. They concluded that HHP at mild temperatures in combination with the addition of nisin may be helpful for improving the safety and preservation of cheeses made from raw milk (30). Similarly, Sudagidan and Yemenicioğlu evaluated the antimicrobial effect of nisin (0.5, 2.5, 12.5, and 25 μg/ml) and lysozyme (1, 2, 3, 4, and 5 mg/ml) on the growth and biofilm formation of 25 strains of *S. aureus* that isolated from cheese (12 strains) and raw milk (13 strains) and figured out that an application of nisin at a level of 25 μg/ml inhibited the growth of all tested strains. In contrast, no inhibition on the growth of tested strain was observed when lysozyme was utilized at levels up to 5 mg/ml. Besides, the results showed that nisin at the growth inhibitory concentration prevented the biofilm formation of strains, while lysozyme was able to inactivate only six strains considerably. Their results showed that the biofilm formation capacity is likely to be affected by the changed bacterial growth kinetics in the presence of lysozyme. They indicated that there might be a potential risk of *S. aureus* growth in dairy products when lysozyme is utilized as a biopreservative (27). Conversely, Amiri et al. studied the antimicrobial characteristics of native and dextran-conjugated lysozyme (400 μg/ml) against *E. coli* and *S. aureus* in cheese curd and found that native and modified lysozyme was effective against *E. coli* at a level of 400 μg/ml in the cheese curd and the glycosylated enzyme was more significant that native enzyme in *E. coli* reduction. Moreover, both lysozyme and modified enzymes were similarly effective against *S. aureus* at 400 μg/ml. As far as we know, lysozyme attacks only glycosidic bonds that presented in the peptidoglycan of bacterial cell wall. On the other hand, due to the presence of hydrophobic material, such as lipopolysaccharide associated with the thin peptidoglycan layer of Gram-negative bacteria, native lysozyme can hardly penetrate and lyse the cell wall of Gram-negative bacteria. Therefore, it is necessary to improve the surface properties of lysozyme. Their results revealed that the improvement of antimicrobial activity of the lysozyme-dextran conjugate against Gram-negative bacteria, such as *E. coli* could be probably ascribed to the excellent surfactant properties and the remaining lytic activity of lysozyme-dextran conjugate. Generally, they stated that due to the improved solubility of lysozyme-dextran conjugate at various temperatures and pH values as well as better surfactant properties in comparison with native enzyme lysozyme, lysozyme-dextran conjugate could considered as a suitable antimicrobial enzyme (62).

Furthermore, Sinigaglia et al. examined the antimicrobial activity of lysozyme (0.25 mg/ml) in combination with various levels of ethylenediaminetetraacetic disodium salt (Na_2_-EDTA) (10, 20, and 50 mmol/L) against spoilage microorganisms, such as coliforms and *Pseudomonadaceae* as well as the functional microbiota of mozzarella cheese during 8 days storage at 4°C. They understood that lysozyme and Na_2_-EDTA could be effective in preventing the growth of coliforms and *Pseudomonadaceae* during 7 days storage. In addition, they reported that the functional microbiota of mozzarella cheese, such as lactic acid bacteria, was not affected by the addition of these active compounds and finally, they concluded that the shelf life of mozzarella cheese could be prolonged by utilizing lysozyme and Na_2_-EDTA in the conditioning brine (59). Similarly, Scharfen et al. investigated the effect of human lysozyme (270 μg/ml) transgenic goat milk on the functionality of lactic acid bacteria in cheese making and found that lysozyme transgenic goat milk did not have an adverse effect on the growth of lactic acid bacteria during cheese-making process and could be potentially used to enhance the characteristics of raw milk cheese production (28). Additionally, Conte et al. investigated the effect of various concentrations of lysozyme (125, 250, and 500 mg/kg) with Na_2_-EDTA (50 mM) with or without modified-atmosphere packaging (MAP) (95:5 CO_2_:N_2_) conditions on the microbiological and sensory quality of burrata cheese during 9 days at 8°C. They found that the combination of lysozyme/Na_2_-EDTA and MAP resulted in a higher microbiological acceptability limit (days) for coliforms and *Pseudomonas* spp. compared with control or the samples containing active substance and stored in the air atmosphere. They stated that the growth of aerobic, Gram-negative bacteria, such as *Pseudomonas* spp. could be decreased by the bacteriostatic effect of CO_2_ that extends the lag phase of tested bacteria. Furthermore, they understood that regardless of the packaging strategy, the cheese’s quality showed decreasing manner during storage. They concluded that lysozyme/Na_2_-EDTA in combination with MAP, especially at the highest lysozyme concentrations (500 mg/kg) could be considered as an effective strategy against spoilage microorganisms (63). Moreover, Doosh et al. examined the effect of hen egg-white lysozyme (250 and 300 mg/kg) on the microbial properties and shelf-life of Iraqi soft cheese made from buffalo milk during 15 days storage at 6°C. They observed that the application of lysozyme prevented the development of total count of bacteria, the count of psychrophilic bacteria, and yeast and mold compared with the untreated sample during 15 days storage. Sensory evaluation showed no significant differences among lysozyme-treated samples and control at zero time, confirming that lysozyme addition had no adverse effect on consumer acceptance. Furthermore, after 3 days of storage lysozyme-treated samples, especially the one containing 300 mg/kg obtained the highest scores in terms of texture, flavor, and bitterness compared with control. These high scores could be attributed to the antimicrobial activity of lysozyme that prevents bacterial growth, particularly psychotropic bacteria, which is responsible for the lipolysis and proteolysis of cheese during storage (25). Additionally, Al-Baarri et al. studied the possibility of shelf-life improvement of Indonesian soft milk cheese (dangke) using lysozyme and lactoperoxidase systems. In this regard, the microbiological properties of dangke samples placed in sterile pure water, lysozyme, lactoperoxidase system (LPS), and a combination of lysozyme and LPS were evaluated during 18 h at room temperature. They figured out that the sample of cheese immersed in sterile pure water had the highest bacterial count at 0 h compared with the treated samples. The lysozyme-treated cheese sample exhibited the lowest total number of bacteria. Furthermore, they found that the microbial growth was inhibited in treated samples for 8 h. The highest antimicrobial activity was observed in the cheese immersed in the combination of LPS and lysozyme followed by lysozyme. They indicated that the antimicrobial activity of lysozyme could be ascribed to its ability to destroy the structure of cell wall bacteria and fungi (64). In a study carried out by Sozbilen and Yemenicioğlu, the effect of pH (4 and 6) and temperature (50 or 60°C for 45 min) on the optimum activity of lysozyme–nisin combination (500–15.6 μg/ml) against *Listeria innocua* in the test buffer was investigated. They understood that heating at pH 4.5 and 6 in combination with lysozyme and nisin resulted in the higher inactivation of *L. innocua* compared with heating alone or heating combined with lysozyme or nisin. Furthermore, they indicated that the antimicrobial activity of lysozyme–nisin combination at 50°C was not pH-dependent. Additionally, they found that milk heating at 50°C preserved 73 and 63% activity of nisin and lysozyme, respectively. They concluded that heating at 50°C in combination with lysozyme and nisin could be applicable as mild treatment in the production of traditional cheese made from raw milk to improve the safety of the cheese and aroma and flavor. Moreover, they reported that the changes of pH range from 2.5–4 to 4.50–6.50 resulted in an increase in the activities of lysozyme from 204–2,039 to 17,705–48,295 U/ml range, respectively. Therefore, to achieve the acceptable antimicrobial properties of lysozyme, the pH of food products should be taken into consideration (29).

Besides the direct usage of lysozyme in cheese or cheese brine, the application of lysozyme as an antimicrobial agent in the packaging, such as film and coating is investigated. Duan et al. examined the antimicrobial activity of chitosan composite film containing lysozyme against various microorganisms inoculated onto the surface of Mozzarella cheese. They observed that chitosan-lysozyme composite films and coatings remarkably decreased the growth of *L. monocytogenes*, *E. coli*, *Pseudomonas fluorescens*, and mold in Mozzarella cheese than laminated films and coatings. This can be ascribed to the higher concentration of chitosan and lysozyme in chitosan-lysozyme composite than in laminated films and coatings. They indicated that beside the peptidoglycan hydrolysis, the non-enzymatic antimicrobial action of lysozyme involving membrane disruption due to the positive charge of lysozyme could be suggested for the antimicrobial properties of lysozyme, especially against Gram- negative bacteria. They claimed that chitosan composite films in combination with lysozyme could be utilized in cheese packaging to control the post-processing microbial contaminants of cheese and therefore, enhance the microbial safety of cheese products (65). Similarly, Mehyar et al. studied the effect of chitosan coating containing lysozyme (60% (w/w) lysozyme/chitosan solution) on the shelf-life, microbial, and sensory properties of Halloumi cheese and found that coating increased the shelf-life of cheese as well as having no adverse effect on the sensory properties of cheese. They concluded that by using chitosan-lysozyme coating in the production of Halloumi cheese, the brine could be reduced from 15 to 10% while the same inhibitory effects against most contaminating microorganisms and shelf-life would be achieved (66).

Additionally, it has been reported that composite edible film containing lysozyme prevented the growth of microorganisms both on the surface and inside the region of Gouda cheese during ripening (67). It has been stated that nano-laminate coating containing lysozyme was able to extend the shelf-life of Coalho cheese and coated cheese sample exhibited lower pH, lipid peroxidation, values of mass loss and microorganisms’ proliferation in comparison with the uncoated sample (68).

Jalilzadeh et al. evaluated the application of lysozyme-xanthan (200, 400, and 500 ppm) gum in whey protein-based edible coating on the shelf life of ultra-filtrated white cheese. They figured out that the growth of *Penicillium chrysogenum*, *E. coli* O157:H7, and *S. aureus* was significantly inhibited by application coating incorporating lysozyme-xanthan gum conjugate in comparison with uncoated cheese and therefore, the shelf life of coated sample increased. Their results showed that lactic acid bacteria were not affected by edible coating during 60 days of ripening. Due to the antimicrobial effect of tested edible film and having no adverse effect on the sensory properties of ultra-filtrated white cheese, they stated that whey protein-based edible coating in combination with lysozyme-xanthan could be applicable in increasing the shelf life of ultra-filtrated cheese (69). Generally, it can be stated that the application of edible film and coating in combination with natural antimicrobial enzymes, such as lysozyme can be considered as an alternative material for the food industry to improve the safety of food products, such as cheese, especially during storage and ripening to prevent post-processing contamination. Table 2 listed selected publications on the application of lysozyme in cheese.

**TABLE 2:** Selected publications regarding the effects of lysozyme addition on the microbial properties of cheese.

Cheese type	The mode of lysozyme application and experiment condition	Lysozyme concentration	Target microorganism	Outcome	References
Model cheeses	Lysozyme added into milk in combination with high hydrostatic pressure (HHP) (60 MPa/210 min/30°C + 400 Mpa/15 min/30°C	(22.4 mg/L)	Spores of *B. cereus* ATCC 9139	Lysozyme did not enhance the sensitivity of the spores to HHP	(30)
Traditional French goat cheese	Human lysozyme transgenic goat milk	270 μg/mL	Lactococci, streptococci, enterococci and *Lactococcus* spp.	Lysozyme transgenic goat milk did not adversely affect the growth of lactic acid bacteria during the cheese-making process. Due to inactivation of lysozyme by pasteurization at 74°C, transgenic technology and lysozyme transgenic goat milk had the potential to improve the safety of raw milk cheese production.	(28)
Mozzarella cheese	Chitosan composite film containing lysozyme storage at 10°C for 14 and 30 days for bacteria and mold, respectively	60% lysozyme in chitosan film-forming solutions	*L. monocytogenes*, *E. coli*, *P. fluorescens*, mold and yeast	Incorporation of 60% lysozyme in chitosan film-forming solutions exhibited greater antimicrobial effect than chitosan alone. The growth of *L. monocytogenes*, *E. coli*, *P. fluorescens* and mold in Mozzarella cheese decreased by application of chitosan-lysozyme composite films and coatings	(65)
Cheese curd	Native, heat- treated and dextran-conjugated lysozyme Ripening at 4°C for 40 days	400 μg/mL	*E. coli* and *S. aureus*	Lysozyme and modified enzymes were effective against *E. coli.* However, the lysozyme-dextran conjugate decreased the populations of *E. coli* by 3 log in cheese curd after 40 days of storage, indicating the improvement of antimicrobial activity of the lysozyme-dextran conjugate against Gram-negative bacteria. Both lysozyme and modified enzymes were similarly effective against *S. aureus* and conjugation with dextran did not increased antimicrobial activity of lysozyme against *S. aureus*.	(62)
Mozzarella cheese	Lysozyme in diluted brine in combination with Na2-EDTA (10, 20 and 50 mmol/L) Storage at 4°C for 8 days	0.25 mg/mL	Total coliforms, *Pseudomonadaceae* and lactic acid bacteria	Lysozyme and Na2-EDTA significantly prevented the growth of coliforms and *Pseudomonadaceae* during the first 7 days of storage. Lactic acid bacteria were not affected by the addition of these active compounds.	(59)
Burrata cheese	Direct addition of lysozyme and Na2-EDTA (50 mM) with or without modified-atmosphere packaging storage at 8°C for 9 days	125, 250 and 500 mg/kg	Total microbial count, psychrotrophic microflora, lactic acid bacilli, yeasts and molds, total coliforms, *Enterobacteriaceae*, *Pseudomonas* spp.	Combination of lysozyme/Na2-EDTA and MAP resulted in higher microbiological acceptability limit (days) for coliforms and Pseudomonas spp. Cheese shelf life was prolonged by the combination of lysozyme/Na2-EDTA and MAP, especially at the highest lysozyme concentration	(63)
Iraqi soft cheese	Addition of lysozyme to the curd of soft cheese. Storage at 6°C for 15 days	250 and 300 mg/kg	Total count of bacteria, psychrophilic bacteria, yeast and mold	Enzyme addition led to a reduction in the development of tested bacteria in comparison to the untreated sample. The higher lysozyme, the better prolonged the shelf life. The highest scores in terms of texture, flavor and bitterness were observed in the sample containing 300 mg/kg lysozyme	(27)
Coalho cheese	Nano-laminate coating containing lysozyme storage at 8°C for 20 days	0.2% (w/v)	Mesophilic and psychotropic microorganisms	The shelf life of Coalho cheese was increased by application of nano-laminate coating containing lysozyme and coated cheese showed lower peroxidation of lipid and microorganisms’ proliferation	(68)
Gouda cheese	Composite edible film whey protein porang flour containing lysozyme ripening for 8 weeks	0, 0.05, and 0.1%	Aerobic plate count, Lactic acid bacteria, *Enterococcus*, Coliform, *E. coli*, *Salmonella*, S. *aureus* and yeast/mold	Native microbial population of coated gouda cheese such as aerobic plate count, lactic acid bacteria, enterococcus, and coliform was not significantly affected by modified lysozyme addition in composite edible. Although the population of native microbial gradually decreased in coated-Gouda cheese, the population of artificial pathogen contamination decreased faster at the beginning of cheese ripening. Generally, composite edible film containing lysozyme was able to prevent the growth of microorganisms both at the surface and inside the region of Gouda cheese during ripening.	(67)
Halloumi cheese	Chitosan coating with or without lysozyme cheese was held at 3°C or 25°C in 5%, 10% or 15% (w/v) NaCl for 18 h.	Lysozyme stock solution was prepared by dissolution at 10% (w/v) in distilled water with the addition of 25% (w/w) glycerol/lysozyme). The lysozyme solution was then mixed with the chitosan solution at a concentration of 60% (w/w) lysozyme/chitosan	Mesophilic, psychrotrophic, anaerobic, LAB, yeasts and molds	Coating increased the shelf-life of cheese without any adverse effect on the sensory properties of cheese. By using chitosan-lysozyme coating, the brine could be reduced from 15% to 10% while the same inhibitory effects against most contaminating microorganisms was obtained. Using chitosan-lysozyme coating in the production of Halloumi cheese, the brine could be reduced from 15 to 10% while the same inhibitory effects against most contaminating microorganisms and shelf-life would be achieved	(66)
Dangke (fresh soft cheese)	Immersion of dangke in various preservation solutions (lysozyme, lactoperoxidase and combination of lactoperoxidase and lysozyme) at 30°C for 10 min storage at 30°C for 18 h	–	Total microbial count	The highest bacterial count at 0 h was observed in the sample immersed in pure sterile water. The combination of lactoperoxidase system and lysozyme could inhibit the growth of microbes in dangke stored for 8 h.	(64)
Ultra-filtrated white cheese	Whey protein-based edible coating containing lysozyme-xanthan gum conjugate. Microbial properties was studied during 28 days storage at 8°C	200, 400, and 500 ppm lysozyme-xanthan conjugate	*P. Chrysogenum*, *E. coli* O157: H7, *S. aureus*, and starter activity	The growth of tested microorganisms was inhibited by application coating incorporating lysozyme-xanthan gum conjugate in comparison to uncoated cheese. Lactic acid bacteria were not affected by edible coating during 60 days of ripening. It has been stated that whey protein-based edible coating could be utilized as a carrier of lysozyme-xanthan to improve the shelf life of ultra-filtrated cheese.	(69)

## Regulatory Status and Toxicology

One of the controversial topics about lysozyme is its role as an allergen. Some studies indicated that lysozyme acts as a weak allergen, while others support the opposite idea (70). As far as we know, egg white is considered allergenic to sensitive individuals, especially children and this sensitivity is related to allergenic proteins in eggs, such as conalbumin (ovotransferrin), ovomucoid, ovalbumin, and lysozyme. Allergic reactions generated by egg white lysozyme were lower in animals and humans than in other egg proteins, such as ovalbumin and albumin (41). Although significant amounts of IgE in the serum of egg-allergic patients have been bonded by lysozyme, the clinical significance of this reaction is unclear (41). It has been reported that lysozyme from egg white has insignificance acute, subacute, and chronic toxicity in animals (23). Furthermore, the long-term consumption of lysozyme as an endogenous food ingredient in eggs, milk as well as the long historic use of lysozyme in cheese that has traditionally been sold over the counter without prepackaging and labeling could be considered as an argument for the safety of lysozyme (23, 41). It has been stated that neither side effects nor immunological responses have been identified in egg-sensitive individuals with or without lysozyme sensitization after consuming lysozyme-containing cheese (71). Therefore, it is likely that the application of egg-white lysozyme in foods at permitted levels could not be considered as a health concern. Lysozyme has been declared as generally recognized as safe (GRAS) by the U.S. Food and Drug Administration (FDA) for use in cheese. Due to the allergenic potential of lysozyme, the FDA has concluded that “Egg white lysozyme” labeling is mandatory for bulk and packaged foods containing lysozyme (72). Additionally, it has been reported by the Joint FAO/WHO Expert Committee on Food Additives (JECFA) that lysozyme is derived from edible animal tissue commonly utilized as food and therefore can be designated as class I enzyme and considered as a food. It has been stated by JECFA that the intake of the low amounts of lysozyme in cheese made from treated milk was not a risk to consumer health (73). It has been approved by many countries to utilize hen egg-white lysozyme to control the growth of spoilage organisms in foods (74). European Additives Directive approved lysozyme as a preservative (E1105) to prevent the late blowing in ripened cheese (23).

## Conclusion

Due to the growing concerns about the applying synthetic additives in food, much effort has been made in the food industry for alternatives to these additives, especially from natural sources to improve the safety and shelf-life of food products. Because of antimicrobial and antibiofilm activity of antimicrobial enzymes, they have been recently considered as a novel natural antimicrobial agent in food preservation. Due to the widespread dispersion of lysozyme in nature and the antimicrobial activity of this enzyme against various bacteria, especially Gram-positive, lysozyme has the potential to be considered a safe food additives in food products. Based on the differences in amino acid sequences and structural properties, lysozymes are categorized into three main types: c-type, g-type, and i-type. Lysozyme from HEWL is considered a major industrial source for practical uses because of its availability and cost-effectiveness. All lysozymes exert antimicrobial activity by hydrolyzing the β-(1,4)-glycosidic linkages between N-acetylmuramic acid (NAM) and N-acetylglucosamine (NAG) of peptidoglycan chains and this activity is affected by various factors, such as ionic strength, presence of lysozyme inhibitors, pH, salt content, water activity, presence of potentiating chemicals, and temperature. Various chemical and physical treatments have been applied to improve the sensitivity of Gram-negative bacteria against lysozyme. Lysozyme is presented as a natural preservative in mammalian milk and therefore, it could be utilized in dairy products, such as cheese and as this study revealed, lysozyme could be applied in cheese making to inhibit the growth of spoilage bacteria and therefore to prevent the texture deterioration, blowing, and unpleasant taste of various cheeses. Moreover, besides the direct usage of lysozyme in cheese or cheese brine, lysozyme could be applied as an antimicrobial agent in the packaging, such as film and coating to preserve its functionality and antimicrobial activity during storage and ripening to prevent post-processing contamination. Generally it could be stated that lysozyme could be considered as an alternative material alone or as part of hurdle technology for the food industry to improve the safety of food products, such as cheese. Additionally, it is suggested to study the application of lysozyme in combination with other antimicrobial enzymes and novel technologies, such as high-intensity pulsed electric field, cold plasma, and irradiation to enhance the antimicrobial efficiency of lysozyme in cheese making in future investigations.

## Author Contributions

MY and NK designed the study. NK, EK, and MK wrote the manuscript. MY, NK, and SS critically revised the manuscript. All authors listed have approved it for publication, contributed to the article, and approved the submitted version.

## Conflict of Interest

The authors declare that the research was conducted in the absence of any commercial or financial relationships that could be construed as a potential conflict of interest.

## Publisher’s Note

All claims expressed in this article are solely those of the authors and do not necessarily represent those of their affiliated organizations, or those of the publisher, the editors and the reviewers. Any product that may be evaluated in this article, or claim that may be made by its manufacturer, is not guaranteed or endorsed by the publisher.
